# The complete chloroplast genome of agave hybrid 11648

**DOI:** 10.1080/23802359.2020.1775145

**Published:** 2020-06-04

**Authors:** Gang Jin, Xing Huang, Tao Chen, Xu Qin, Jingen Xi, Kexian Yi

**Affiliations:** a Guangxi Subtropical Crops Research Institute, Nanning, PR China; b Environment and Plant Protection Institute, Chinese Academy of Tropical Agricultural Sciences, Haikou, PR China; c Key Laboratory of Integrated Pest Management on Tropical Crops, Ministry of Agriculture and Rural Affairs, Haikou, PR China; d Hainan Key Laboratory for Monitoring and Control of Tropical Agricultural Pests, Haikou, PR China

**Keywords:** *Agave* H11648, chloroplast genome, phylogenetic tree

## Abstract

*Agave* hybrid 11648 is the most widely cultivated agave variety for sisal fiber production around the world. In the present study, we have successfully sequenced the chloroplast genome of *A*. H11648. The complete chloroplast genome size is 157,274 bp in length with a GC content of 37.8%. The genome contains a large single copy region (LSC) of 85,896 bp, a small single copy region (SSC) of 18,230 bp, and a pair of inverted repeat regions (IR) of 26,574 bp. 121 genes are annotated in the chloroplast genome. The numbers of protein-coding, tRNA and rRNA genes are 99, 40 and 8, respectively. Phylogenetic tree reveals that *A*. H11648 is closely related to *A. americana*.

Sisal, an important kind of nature fiber with the properties of tough texture, high strength and friction resistance, has been widely used in navigation, automotive, papermaking, etc. (Li et al. 2000). As the main cultivar, *A*. H11648 ((*A. amanuensis* × *A. angustifolia*) × *A. amaniensis*) has been widely cultivated in tropical areas of Africa, South America and China (Robert et al. 2008). According to a previous study, a phylogenetic tree has been constructed with the partial chloroplast (cp) sequences of four agave species (Huang et al. 2018). However, the systematic position and phylogenetic relationship of *A*. H11648 still remain ambiguous at cp genome level. In the present study, we assembled the complete cp genome of *A*. H11648 with next-generation sequencing, with the aim to reveal the phylogenetic relationship of agave species.

The leaves of *A*. H11648 were collected the experimental field (22.90°N, 108.33°E) of Guangxi Subtropical Crops Research Institute, Nanning, China. The modified CTAB method was used to extract the total genomic DNA (Doyle and Doyle 1987). The specimen was deposited in Herbarium of Guangxi Subtropical Crops Research Institute (HGS-jm2018004). Total DNA was sent to Sangon Biotech (Shanghai, China) for library construction and next-generation sequencing. A total of 1.29 Gb short reads data was generated using Illumina HiSeq 2500 platform. The trimmed reads were selected for cp genome assembly by SPAdes software (Bankevich et al. 2012). The assembled cp genome was annotated by DOGMA (Wyman et al. 2004), which was corrected with Geneiousv11.0.3 (Kearse et al. 2012). The complete cp genome sequence was deposited in GenBank under the accession number MG642741.

The complete chloroplast genome size is 157,274 bp in length with a GC content of 37.8%. The genome contains a large single copy region (LSC) of 85,896 bp, a small single copy region (SSC) of 18,230 bp, and a pair of inverted repeat regions (IR) of 26,574 bp. 121 genes are annotated in the chloroplast genome. The numbers of protein-coding, tRNA and rRNA genes are 99, 40 and 8, respectively. Among these, ten genes (*trnA-UGC*, *trnI-GAU*, *ndhB*, *ycf15*, *rpl2*, *trnK-UUU*, *atpF*, *rpoC1*, *trnL-UAA* and *trnV-UAC*) contains one intron and three with two introns (*ycf68*, *ycf3* and *clpP*).

The cp genome sequences of 26 species were selected for phylogenetic analysis (23 species in Agavoideae and 3 species as outgroup including *Albuca kirkii*, *Nolina atopocarpa* and *Oziroe biflora*) (McKain et al. 2016; Lee et al. 2019). The result indicated that *A*. H11648 is closely related with *A. americana* (Figure 1), which was consistent with our previous study (Huang et al. 2018). The cp genome sequences were aligned using MAFFT (Katoh and Standley 2013). The phylogenetic tree was constructed in MEGA7 software with the neighbor-joining method with 10000 bootstrap replicates (Kumar et al. 2016). This study will benefit future studies related to chloroplast in *Agave* genus.

**Figure 1.**
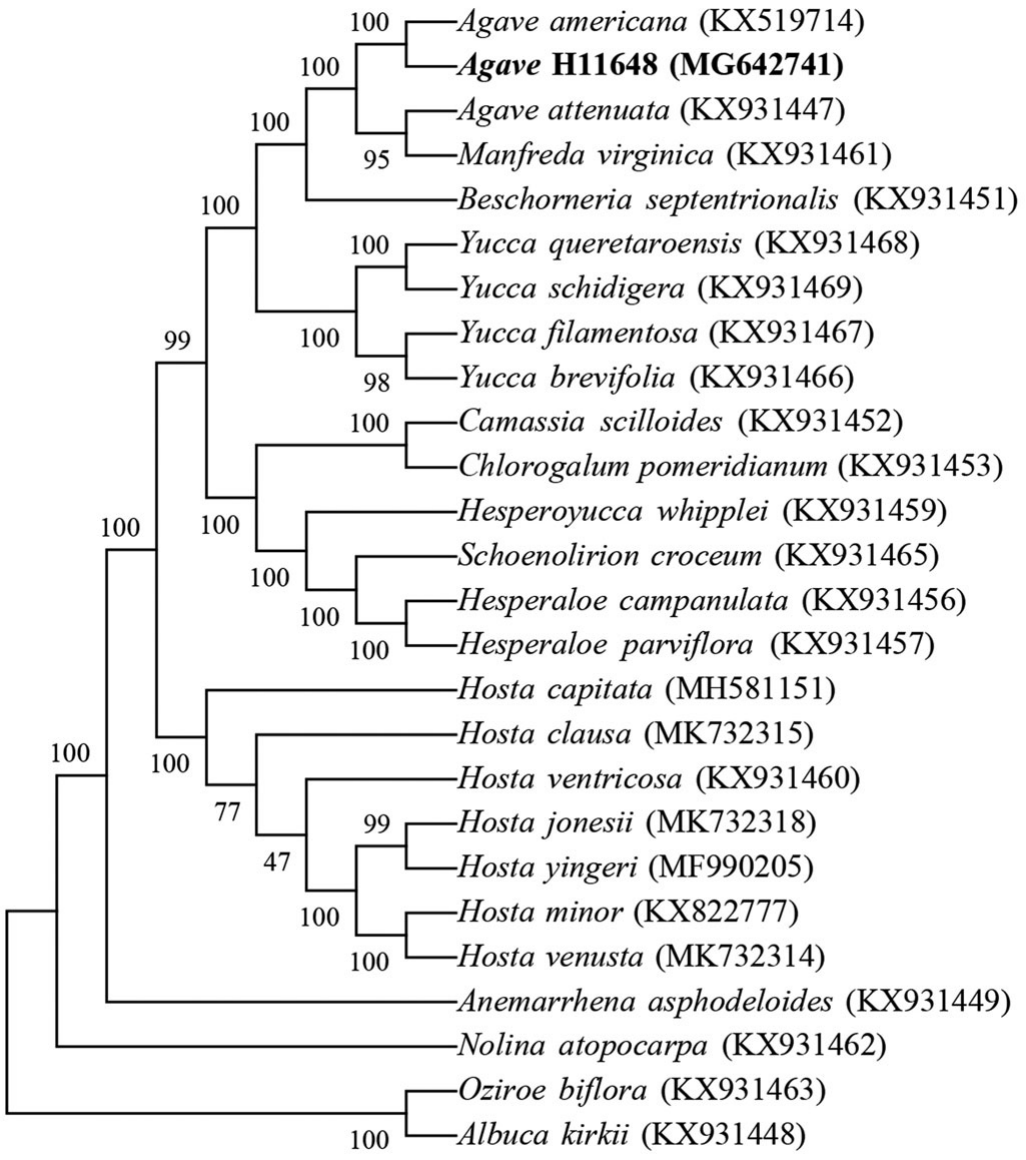
Phylogenetic tree of 26 chloroplast genomes.

## Data Availability

The data that support the findings of this study are fully available in GenBank (https://www.ncbi.nlm.nih.gov/nuccore/MG642741).
